# Rewiring the Metabolic Network to Increase Docosahexaenoic Acid Productivity in *Crypthecodinium cohnii* by Fermentation Supernatant-Based Adaptive Laboratory Evolution

**DOI:** 10.3389/fmicb.2022.824189

**Published:** 2022-03-02

**Authors:** Liangsen Liu, Jinjin Diao, Yali Bi, Lei Zeng, Fangzhong Wang, Lei Chen, Weiwen Zhang

**Affiliations:** ^1^Laboratory of Synthetic Microbiology, School of Chemical Engineering and Technology, Tianjin University, Tianjin, China; ^2^Key Laboratory of Systems Bioengineering, Ministry of Education of China, Tianjin, China; ^3^Collaborative Innovation Center of Chemical Science and Engineering, Tianjin, China; ^4^Center for Biosafety Research and Strategy, Tianjin University, Tianjin, China; ^5^Law School, Tianjin University, Tianjin, China

**Keywords:** adaptive laboratory evolution, *Crypthecodinium cohnii*, growth, DHA content, fermentation supernatant, quantitative proteomics

## Abstract

Docosahexaenoic acid (DHA, 22:6n-3) plays significant roles in enhancing human health and preventing human diseases. The heterotrophic marine dinoflagellate *Crypthecodinium cohnii* is a good candidate to produce high-quality DHA. To overcome the inhibition caused by the fermentation supernatant in the late fermentation stage of DHA-producing *C. cohnii*, fermentation supernatant-based adaptive laboratory evolution (FS-ALE) was conducted. The cell growth and DHA productivity of the evolved strain (FS280) obtained after 280 adaptive cycles corresponding to 840 days of evolution were increased by 161.87 and 311.23%, respectively, at 72 h under stress conditions and increased by 19.87 and 51.79% without any stress compared with the starting strain, demonstrating the effectiveness of FS-ALE. In addition, a comparative proteomic analysis identified 11,106 proteins and 910 differentially expressed proteins, including six stress-responsive proteins, as well as the up- and downregulated pathways in FS280 that might contribute to its improved cell growth and DHA accumulation. Our study demonstrated that FS-ALE could be a valuable solution to relieve the inhibition of the fermentation supernatant at the late stage of normal fermentation of heterotrophic microalgae.

## Introduction

Docosahexaenoic acid (DHA, 22:6n-3), a long-chain polyunsaturated fatty acid (PUFAs) and a structural constituent of membranes, specifically in components of the nervous system, such as the brain and retina (Ferraro et al., 2013), is essential for human beings, because they are poor DHA synthesizers (Muskiet et al., 2004). In addition, DHA plays significant roles in enhancing human health and preventing human diseases, such as hypertension, diabetes, and cancers (Horrocks and Yeo, 1999). Marine fishes are the traditional source of DHA; however, the supply of marine fishes depends on gradually diminishing sea fishing, and fish-based DHA has obvious disadvantages, such as heavy metal contamination, the typical fishy smell, and the presence of eicosapentaenoic acid (EPA) (Mendes et al., 2008). Currently, the heterotrophic marine dinoflagellate *Crypthecodinium cohnii* is a good candidate due to its ability to produce high-quality DHA with less than 1% of other types of PUFAs and without any EPA content (Liu et al., 2018). The lipid content of *C. cohnii* can reach more than 50% of its dry cell weight (DCW), and the proportion of DHA in total fatty acids (TFAs) can be approximately 40–50% (Diao et al., 2019).

The cost of DHA production largely depends on the overall volumetric productivity in *C. cohnii* (Lolke et al., 1998; De Swaaf et al., 2001). In recent years, significant efforts have been made to improve DHA productivity in *C. cohnii* using approaches such as optimizing fermentation conditions (Liu et al., 2018), employing different carbon sources (De Swaaf et al., 2003a,b), and enhancing strain improvement using the acetyl-CoA carboxylase inhibitor sethoxydim (Liu et al., 2017). However, certain bottlenecks remain, such as low tolerance to the inhibitory fermentation supernatant at the late stage of fermentation, which significantly limits further improvements of DHA productivity (Liu et al., 2020). It has been previously reported that the fermentation supernatant, in which the accumulation of unspent medium components or metabolites exported by *C*. *cohnii* at the late stage of fermentation occurs, could inhibit the growth and DHA synthesis in *C*. *cohnii* (Liu et al., 2020). Similar inhibition phenomena of the fermentation supernatant were also observed in the cultivation of many other microbes (Liu et al., 2002; Zhang et al., 2013, 2016; Mazzoleni et al., 2015; Sha et al., 2019). For example, soluble algal products from 6.4–25.8 mg/L in the fermentation supernatant produced by *Scenedesmus* sp. LX1 reduced the maximum algal density by 50–80% (Zhang et al., 2013). The self-produced inhibitory compounds in the fermentation supernatant resulted in a growth decline during fed-batch cultures in *Saccharomyces cerevisiae* (Mazzoleni et al., 2015). In addition, the fermentation supernatant from the late growth phase was found to be a stronger growth inhibitor of *Scenedesmus acuminatus* (Sha et al., 2019). Moreover, the classification of the specific inhibitory compounds in the fermentation supernatant is usually challenging because of its significant complexity (Zhang et al., 2013, 2016; Mazzoleni et al., 2015; Liu et al., 2020). Therefore, to date, very few studies have been conducted regarding the inhibition relief of the fermentation supernatant at the late stage of normal fermentation, especially in *C*. *cohnii*.

Adaptive laboratory evolution (ALE), which subjects microbes to serial or continuous cultivation for multiple generations, has been demonstrated to be an effective approach to achieve improved inhibitor tolerance (Kildegaard et al., 2014; Shui et al., 2015; Diao et al., 2019). For example, ALE was used to improve the tolerance to sethoxydim in *C. cohnii*, increasing carbon equivalents from starch to lipids (Diao et al., 2019). Specific inhibitory compounds in the fermentation supernatant are not clear in *C. cohnii*; hence, fermentation supernatant-based adaptive laboratory evolution (FS-ALE) using the inhibitory fermentation supernatant as a selective stress might relieve the inhibitory effects of the fermentation supernatant at the late stage of normal fermentation in *C*. *cohnii*. Proteomics analyses have been widely used to determine the underlying mechanisms of ALE (César et al., 2018; Papanek et al., 2018; Strucko et al., 2018). More importantly, recent technological breakthroughs allow comparative proteomics analyses even when the genomic information of the studied species is not available (Zhang et al., 2015), making it a suitable tool to decipher the underlying mechanisms of tolerance in *C*. *cohnii*, whose full genome sequences are unavailable.

In this study, to improve the DHA productivity of *C. cohnii*, FS-ALE was developed by gradually increasing the amount of the inhibitory fermentation supernatant collected from fed-batch fermented *C. cohnii* ATCC 30556 (starting strain) at the stationary growth phase in the regular fermentation medium. This study provides an innovative solution (FS-ALE) to relieve the inhibition of the fermentation supernatant at the late stage of normal fermentation in *C. cohnii.* Furthermore, quantitative proteomics was employed for the first time for *C. cohnii* to identify the rewired metabolic network and increase DHA productivity in *C. cohnii*. This study also provides valuable insights for understanding the mechanisms of cell growth and lipid accumulation in *C. cohnii*.

## Materials and Methods

### Strain and Maintenance

*Crypthecodinium cohnii* ATCC 30556 was maintained in a dark static culture of basal medium containing 9 g/L glucose (Jiang Tian, China), 25 g/L sea salt (Sigma-Aldrich, United States), and 2 g/L yeast extract (OXOID, United Kingdom) at 25°C (Sui et al., 2014). *C. cohnii* was cultivated for 2 days at 25°C, pH 6.5 and 180 rpm in basal medium; and subcultivated 3 times before being used as a seed.

### Fermentation Supernatant-Based Adaptive Laboratory Evolution

The fermentation supernatant was collected from the fed-batch fermented *C. cohnii* ATCC 30556 (starting strain) with a pulse-feeding nitrogen strategy at a constant C/N (*w/w*) ratio of 4:1, the same method used in our previous study (Liu et al., 2018). At the end of the fermentation that corresponded to the stationary growth phase, the fermentation broth with the glucose concentration adjusted to 27 g/L was harvested by centrifugation (17696 × *g*, 20 min, and 4°C), and the aqueous phase was collected as the fermentation supernatant under ensured sterile operation. Then, the fermentation supernatant was filtered through a 0.45 μm membrane and stored at 4°C before use.

FS-ALE was conducted by successive subcultivation of *C. cohnii* ATCC 30556 (wild-type strain) using medium amended with varying contents of the fermentation (30–90%) supernatant as the selective pressure. The initial inoculation densities were all controlled to 0.1 (OD_490_). *C. cohnii* ATCC 30556 was subcultivated for 3 days at 25°C and 180 rpm in regular fermentation medium (27 g/L glucose, 25 g/L sea salt, and 6 g/L yeast extract) amended with the fermentation supernatant. The initial percentage of the fermentation supernatant in the medium was set to 30% (*v*/*v*) and was gradually increased to 90% (*v*/*v*) throughout the FS-ALE process, with a 5% increase for each gradient of the supernatant content. So the fermentation broth was consisted of normal medium and fermentation supernatant in proportions ranging from 30 to 90%. Within each percentage of the fermentation supernatant in the medium, the evolved strain was subcultivated for 20 cycles. The whole adaptive evolution process went through 280 propagation cycles with 3 days per cycle, corresponding to 840 cultivation days. The endpoint-evolved strain was named *C. cohnii* FS280 and was selected for further phenotypic characterization and proteomics analysis.

### Batch Culture of Shake Flasks and Bioreactors for Phenotypic Verification

For batch culture in shake flasks, *C. cohnii* ATCC 30556 (WT) and *C. cohnii* FS280 (FS280) were cultivated (25°C and 180 rpm) in medium mixed with the fermentation supernatant and regular fermentation medium (27 g/L glucose, 25 g/L sea salt, and 6 g/L yeast extract), with fermentation supernatant ratios of 30, 60, and 90% (*v/v*), respectively.

For the batch culture in the bioreactor, WT and FS280 were cultivated in 5-L bioreactors (Demei, China) containing 3 L medium (81 g/L glucose monohydrate, 25 g/L sea salt, and 12 g/L yeast extract). The seeds were cultured at 25°C and 180 rpm in regular fermentation medium for 84 h. The inoculum size was 1.8 g/L (DCW), and dissolved oxygen was maintained above 30% oxygen saturation. H_2_SO_4_ and antifoam SE-15 were used to control the pH at 6.5 ± 0.1 and foam, respectively.

### Analyses of Cell Density, Total Nitrogen, Dry Cell Weight, Glucose Concentration, Ammonium Nitrogen Concentration, Total Lipids, Specific Growth Rate, and DHA Productivity

Cell density was measured by an ELx808 Absorbance Microplate Reader (BioTek, United States) or UV-1750 spectrophotometer (Shimadzu, Japan) at OD_490_. Total nitrogen was estimated by a K9860 Kjeldahl Analyzer (Hanon Instruments, China). DCW, glucose concentration, ammonium nitrogen concentration, total lipids and DHA were determined according to methods described in our previously published study (Liu et al., 2018). Since yeast extract is a mixture, ammonium nitrogen concentration, the determination method of which is well established and widely used for the measurements in the fermentation broth, was used to characterize nitrogen concentration during the fermentation process. DHA was analyzed by an Agilent 5975 MSD/7890 instrument according to previous study (Liu et al., 2018). The specific growth rate (μ) in the exponential phase is expressed as μ = (ln X_2_-ln X_1_)/(t_2_-t_1_), where X represents the OD_490_ or DCW at time t (Liu et al., 2015). DHA productivity = total DHA/(culture volume × fermentation time).

### Analyses of Intracellular Carotenoids, Starches, and Proteins

Intracellular carotenoids were analyzed according to a previous method with minor modifications (Sun et al., 2019). Briefly, 20 mg freeze-dried algae powder was ground using a mortar and pestle in liquid nitrogen and then extracted three times with acetone. The extraction system was allowed to stand in an icebox for 5 min. Then, the extract was dried in a vacuum concentrator after centrifugation. When the extract completely volatilized, 100 μL acetone was added to dissolve the carotenoids. The supernatant was collected for detection by an Agilent 1260 series binary HPLC system.

Intracellular starches were determined as described in our previous study (Diao et al., 2019). For intracellular proteins, 5 mg freeze-dried algae powders were used to extract total proteins with an Algae Protein Extraction Kit (BestBio, China) according to the manufacturer’s instructions. Similarly, the total protein concentration was determined by the BCA Protein Quantitation Assay (KeyGEN BioTECH, China) according to the manufacturer’s instructions.

### iTRAQ Quantification Proteomics Analysis

Cell samples of WT and FS280 were collected (3000 × *g*, 10 min and 4°C) at 72 h corresponding to the exponential growth phase, which were cultivated (25°C and 180 rpm) in medium mixed with the fermentation supernatant (90% *v/v*) and regular fermentation medium (10% *v/v*). Samples were washed with phosphate-buffered saline (pH 6.5) 3 times, frozen immediately using liquid nitrogen after centrifugation (239 × *g*, 10 min and 4°C) and then kept at -80°C for subsequent RNA extraction and protein extraction.

*De novo* transcriptomic analysis of FS280 and WT was performed based on a protocol published previously by our group (Pei et al., 2017). The amino acid sequences translated from the coding sequence (CDS) of the obtained *de novo* transcriptomic analysis were used as the protein database for the following iTRAQ proteomics analysis.

Protein extraction and LC-MS/MS proteomic analysis were conducted at the Beijing Genomics Institute (BGI) (Shenzhen, China). Briefly, (i) for protein preparation, and the concentration detection and sample integrity test were conducted with the Bradford assay and polyacrylamide gel electrophoresis, respectively. Each sample with 100 μg protein was digested with Trypsin Gold (Promega, Madison, WI, United States) at a protein:trypsin ratio of 40:1 at 37°C overnight after sample quality assessment. Peptide labeling (WT-1 117, WT-2 118, FS280-1 119, FS280-2 121) and peptide fractionation were performed using the iTRAQ Reagent 8-plex Kit and Shimadzu LC-20AB HPLC Pump system, respectively; (ii) for LC-MS/MS proteomic analysis, the fraction was resuspended in buffer A after vacuum drying, and the supernatant was loaded onto Thermo Fisher Scientific™ UltiMate™ 3000 UHPLC system after centrifuging (20,000 × *g* and 10 min). Mass spectrometer detection was conducted by tandem mass spectrometry QEXACTIVE HF X (Thermo Fisher Scientific, San Jose, CA, United States) for data-dependent acquisition detection via nanoelectrospray ionization after the peptides were separated by nanoHPLC; and (iii) for bioinformatics analysis, the amino acid sequences from the CDS of the *de novo* transcriptomic analysis were used as the protein database. The raw data were converted into mgf files through the Thermo Fisher Scientific tool Proteome Discoverer, and Mascot (version 2.3.02) was used to search the mgf files in the transcriptome database to identify proteins with at least one unique peptide. IQuant was used to analyze the labeled peptides quantitatively (Wen et al., 2014). The protein quantification process was conducted as follows: protein identification, tag impurity correction, data normalization, missing value imputation, protein ratio calculation, statistical analysis, and results presentation. The peptide-spectrum matches (PSMs) and protein false discovery rate (FDR) were both estimated at a FDR level of 1% to provide reliable significance detection (Savitski et al., 2015). WT-1/FS280-1 and WT-2/FS280-2 were used as the comparison group, and the final differentially expressed proteins were defined as at least one replicate that was changed by more than 1.2-fold and had a *Q*-value less than 0.05 (Zhu et al., 2009; Zhang et al., 2015). The coefficient of variation (CV), the ratio of the standard deviation to the mean, was used to evaluate reproducibility.

The mass spectrometry proteomics data have been deposited to the ProteomeXchange Consortium^1^ via the iProX partner repository (Ma et al., 2019) with the dataset identifier PXD030028.

### Statistical Analysis

All experiments in shake flasks were conducted in three biological replicates. All data are presented as the mean ± standard deviation. The data were evaluated by Student’s *t*-test (*p < 0.05, ^**^p < 0.01, and ^***^p < 0.001).

## Results and Discussion

### Fermentation Supernatant-Based Adaptive Laboratory Evolution to Alleviate the Inhibition of Growth and Docosahexaenoic Acid Synthesis in *Crypthecodinium cohnii*

**FIGURE 1:**
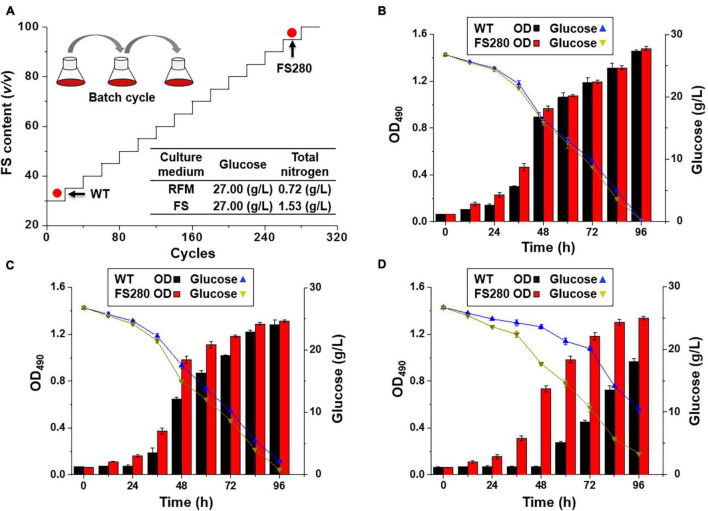
Fermentation supernatant-based adaptive laboratory evolution. **(A)** The process of ALE: RFM, regular fermentation medium; FS, fermentation supernatant; WT, *C. cohnii* ATCC 30556; FS280, *C. cohnii* FS280. **(B)** Cultivation with 30% (*v/v*) fermentation supernatant. **(C)** Cultivation with 60% (*v/v*) fermentation supernatant. **(D)** Cultivation with 90% (*v/v*) fermentation supernatant.

Our previous results showed that more than 20% (*v*/*v*) fermentation supernatant could inhibit the growth and DHA synthesis in *C*. *cohnii* (Liu et al., 2020). To improve tolerance to the inhibitory fermentation supernatant at the late stage of fermentation in *C*. *cohnii*, FS-ALE was developed by successive subcultivation. We used a regular fermentation medium amended with varying contents of the fermentation supernatant collected from fed-batch fermented *C. cohnii* ATCC 30556 at the stationary growth phase as a selective pressure. The purpose of this procedure was to enrich the population with enhanced tolerance to the inhibitory fermentation supernatant (Figure 1A). The glucose concentrations in the regular fermentation medium and the fermentation supernatant were the same, 27 g/L, but the total nitrogen concentration in the fermentation supernatant was increased by 112.50% compared with that in the regular fermentation medium (Figure 1A).

Our previous studies suggested that 30% (*v/v*) fermentation supernatant could result in the inhibition of the growth and DHA synthesis in *C. cohnii* (Liu et al., 2020), and similar phenomena were also observed in other microbes (Yin et al., 2018). For example, the growth of *Schizochytrium* sp. was inhibited by more than 20% (*v/v*) fermentation supernatant, possibly due to the increased toxic components in the presence of a percentage of the fermentation supernatant (Yin et al., 2018). The inhibitory compounds might be from the accumulation of unspent medium components or organic matter, such as proteins, lipids, carbohydrates, fatty acids, and humic substances, that cells release and are difficult to fully classify (Zhang et al., 2016; Sha et al., 2019; Liu et al., 2020). Therefore, the fermentation supernatant was directly used as the selection pressure for ALE.

### Comparative Characterization Between FS280 and WT Under Stress From the Fermentation Supernatant

The evolved *C. cohnii* FS280 and WT were cultivated in regular fermentation medium amended with 30, 60, and 90% (*v/v*) fermentation supernatant (Figures 1B–D). The specific growth rates of FS280 between 0 h and 48 h with 30 and 60% (*v/v*) fermentation supernatant were increased by 2.73 and 21.75% compared with those of WT, respectively (Figures 1B,C). The specific growth rate of FS280 between 0 h and 48 h with 90% (*v/v*) fermentation supernatant was 0.0511 h^–1^, whereas WT did not grow until 60 h under this condition (Figure 1D). The results showed that FS280 was able to grow better than WT under all the stress conditions (Figures 1B–D), indicating the improved tolerance of FS280 to the fermentation supernatant compared to WT. Moreover, the improved tolerance was fermentation supernatant content dependent. The growth of FS280 was increased by 161.87 and 38.39% at 72 h and 96 h, respectively, under 90% (*v/v*) fermentation supernatant compared with that of WT (Figure 1D). The residual glucose during the fermentation process was also monitored, which was consistent with the differential growth (OD_490_) of the strain under different conditions (Figures 1B–D).

**FIGURE 2:**
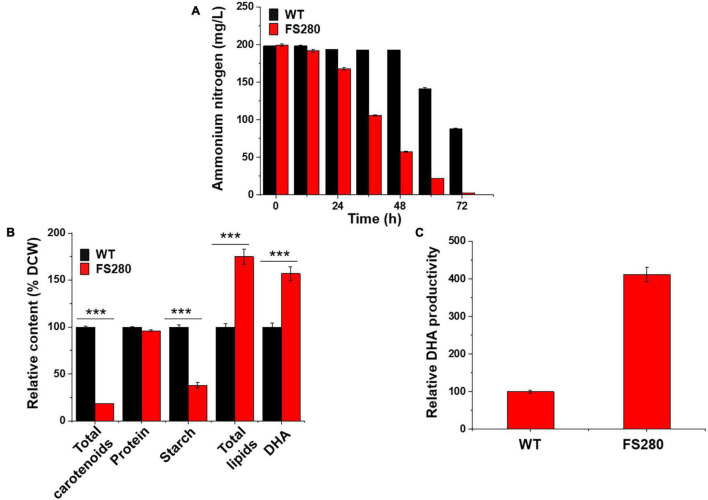
Comparative characterization between FS280 and WT in the presence of 90% (*v/v*) fermentation supernatant. **(A)** The changes in the ammonium nitrogen concentration over 72 h. **(B)** Relative content of cell components at 72 h: DCW, dry cell weight. **(C)** Relative DHA productivity at 72 h (the biomass was expressed by OD_490_). **p < 0.01 and ***p < 0.001.

Further characterization of FS280 and WT in the presence of 90% (*v/v*) fermentation supernatant was conducted. As shown in Figure 2A, ammonium nitrogen in FS280 was almost fully consumed at 72 h, whereas in WT, it only approximately half of the starting concentration was consumed at the same time. This result was consistent with the growth difference of the two strains. Seventy-two hour cells corresponding to the exponential growth phase in the presence of 90% (*v/v*) fermentation supernatant were selected to explore the underlying mechanisms of the enhanced tolerance of FS280. The total lipid content in FS280 was increased by 74.91% compared with that in WT (Figure 2B). At the same time, the content of DHA in FS280 was increased by 57.03% compared with that in WT (Figure 2B). Notably, compared with WT, the DHA productivity in the evolved strain FS280 was increased by 311.23% with the addition of 90% (*v/v*) fermentation supernatant to the medium at 72 h (Figure 2C), indicating the improved performance of FS280 for DHA production. FS-ALE was demonstrated to be an effective method to relieve the inhibition of the fermentation supernatant, which also could be used for recycling the fermentation supernatant that was limited by the presence of complex inhibitors (Wang et al., 2018).

The total starches, proteins and carotenoids in both strains at 72 h showed that the content of total starches in FS280 was only 38.23% (*p* < 0.001) of that in WT and the content of total carotenoids in FS280 was only 18.38% (*p* < 0.001) of that in WT, while the protein content in the evolved strain FS280 was 96.19% of that in WT, with no significant difference (Figure 2B). The above results suggested that the enhanced total lipid content in FS280 might be at the expense of starches and carotenoids, and the metabolic flux might be redirected to the biosynthesis of lipids from starches and carotenoids. Consistent with our results, light illumination was efficient in increasing the TFAs content in *Crypthecodinium* sp. SUN, mainly at the expense of starches (Sun et al., 2017). It was reported that slightly inhibited TFAs biosynthesis could increase the carotenoid yield in *Crypthecodinium* sp. SUN, likely due to the redirection of NADPH and ATP from TFAs to carotenoids, suggesting the competitive relationship of NADPH and ATP between TFAs and carotenoid biosynthesis (Sun et al., 2019). Therefore, the increased lipids found in this study might partially be due to the redirection of NADPH and ATP from carotenoids to TFAs.

### Comparative Characterization Between FS280 and WT Without the Fermentation Supernatant

**FIGURE 3:**
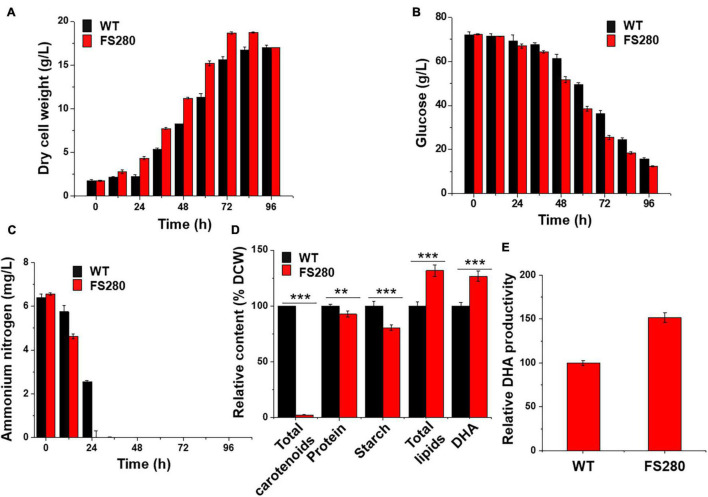
Comparative characterization between FS280 and WT without the fermentation supernatant. **(A)** Changes in dry cell weight. **(B)** changes in glucose concentration. **(C)** Changes in ammonium nitrogen concentration. **(D)** Relative content of cell components at 72 h. **(E)** Relative DHA productivity at 72 h. **p < 0.01 and ***p < 0.001.

The growth of FS280 and WT was compared in fermentation medium (81 g/L glucose monohydrate, 25 g/L sea salt, and 12 g/L yeast extract) in 5-L bioreactors to evaluate whether FS280 had better DHA production in the fermentation medium without the inhibitory fermentation supernatant. The results showed that the growth of FS280 was significantly increased compared with that of WT, with a DCW 19.87% greater than that of WT at 72 h (Figure 3A). The amount of glucose and ammonium nitrogen consumption during the batch culture of the bioreactor confirmed the improved growth of FS280 (Figures 3B,C). Ammonium nitrogen in FS280 was fully consumed at 24 h, while ammonium nitrogen in WT remained at approximately 40% of the starting concentration at 24 h (Figure 3C). Similar to the above results in the presence of 90% (*v/v*) fermentation supernatant, the content of total lipids in FS280 was increased by 31.73% and the content of DHA in FS280 were increased by 26.63% compared with those in WT at 72 h (Figure 3D). The DHA productivity at 72 h of FS280 was increased by 51.79% compared with that of WT (Figure 3E). The content of starches in FS280 was only 80.61% of that in WT (*p* < 0.001), and the content of carotenoids in FS280 was only 2.46% of that in WT (*p* < 0.001), while the protein content in FS280 was reduced slightly and was 92.86% of that in WT (*p* < 0.01) at 72 h (Figure 3D), suggesting that the improved total lipid accumulation in FS280 might be at the expense of starches, carotenoids and proteins.

The evolved strain FS280 obtained by FS-ALE with improved tolerance to the inhibitory fermentation supernatant was demonstrated to have improved DHA productivity compared with WT, whether in the presence of 90% (*v/v*) fermentation supernatant or under normal fermentation conditions without the fermentation supernatant stress (Figures 2C, 3E). The inhibitory compounds in the fermentation supernatant influenced the growth of microorganisms, especially at the late stage of normal fermentation (Xue et al., 2010; Pinhal et al., 2019), and also restricted the recycling of the fermentation supernatant (Zhang et al., 2016; Sha et al., 2019). Studies have been conducted to overcome the inhibitory problems of the fermentation supernatant (Xue et al., 2010; Wang et al., 2018; Sha et al., 2019). For example, repeated fed-batch feeding was used to avoid inhibition at the late stage of normal fermentation by ending the fermentation cycle early and starting a new cycle (Xue et al., 2010). Similarly, the addition of granular activated carbon to remove the inhibitors and UV-based photolysis processes to degrade the inhibitors were used to treat the fermentation supernatant to allow it to be recycled (Wang et al., 2018; Sha et al., 2019). However, few studies have been conducted to solve the inhibition at the late stage of a normal fermentation. FS-ALE provided an innovative approach to address the inhibition at the late stage of normal fermentation, and the inhibition of recycling the fermentation supernatant was solved simultaneously.

### Comparative Proteomic Analysis of FS280 and WT in the Presence of 90% (*v/v*) Fermentation Supernatant

iTRAQ-based comparative proteomics analysis was employed to investigate the mechanisms of the improved growth of and enhanced total lipid accumulation in the evolved strain FS280 in the presence of 90% (*v/v*) fermentation supernatant compared with those in WT. Although *de novo* transcriptomic analyses were conducted for the DHA-producing *C. cohnii* during fed-batch fermentation in our previous study (Pei et al., 2017), considering the possibility of mutations occurring during the total 840 days of evolution in FS280, *de novo* transcriptomic analyses of FS280 and WT were also performed in this study. The amino acid sequences from the CDS were used as the protein database. A total of 87,562 unigenes were identified in the *de novo* transcriptomic analysis, of which 61.16% were annotated in the database (NR, NT, SwissProt, KEGG, KOG, Pfam, and GO), similar to our previous study (82,106 unigenes) (Pei et al., 2017). For the iTRAQ-based comparative proteomic analysis, two replicates of strains FS280 (FS280-1 and FS280-2) and WT (WT-1 and WT-2) were collected at 72 h corresponding to the exponential phase of growth in the presence of 90% (*v/v*) fermentation supernatant and were subjected to the following LC-MS/MS proteomic analysis.

#### Overview of the Proteomic Analysis

According to the iTRAQ proteomic analysis, a total of 61,238 peptides corresponding to 11,106 proteins were identified with 1% FDR (Supplementary Figures 1A–C and Supplementary Table 1A). Although the genome sequence of *C. cohnii* is not currently available, the number of identified proteins in this proteomic analysis was greater than those reported in other dinoflagellates, such as *Prorocentrum donghaiense* (7,731), *Alexandrium catenella* (6,577), and *Pyrocystis lunula* (3,182) (Fajardo et al., 2019; Zhang H. et al., 2019; Zhang S. F. et al., 2019). The unique peptide number, protein coverage and protein mass distribution are indicated in Supplementary Figure 1. A total of 68.63% of the identified proteins had at least two unique peptides (Supplementary Figure 1A), and 52.31% of the identified proteins had a protein coverage higher than 10% (Supplementary Figure 1B). The weight of most proteins was distributed between 30–40 kDa and 40–50 kDa (Supplementary Figure 1C). The above results of the whole proteome distribution were similar to the iTRAQ-based quantitative proteomic analysis of a toxigenic dinoflagellate, which provided the first comprehensive dataset of the dinoflagellate proteome (Zhang et al., 2015).

Based on the KEGG database, all the identified proteins were divided into 20 functional classifications in 6 branches of the metabolic pathway (Supplementary Figure 2). It was interesting to find that a total of 163 (1.47%) proteins were classified into an environmental adaptation that might be associated with fermentation supernatant stress, which was not found in other dinoflagellates facing environmental stress (Zhang et al., 2018).

#### Differentially Expressed Proteins

**FIGURE 4:**
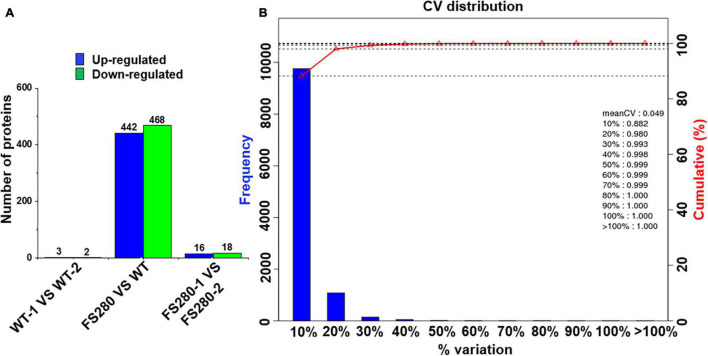
Protein quantification in *C. cohnii*. **(A)** Histogram of differentially expressed proteins. **(B)** Distribution of the CV (coefficient of variation) in replicates. The CV is defined as the ratio of the standard deviation to the mean. The *X*-axis represents the deviation among the protein ratios of the repetitive samples. The *Y*-axis represents the percentage of proteins at a certain angle that comprised the quantified amount of protein.

**FIGURE 5:**
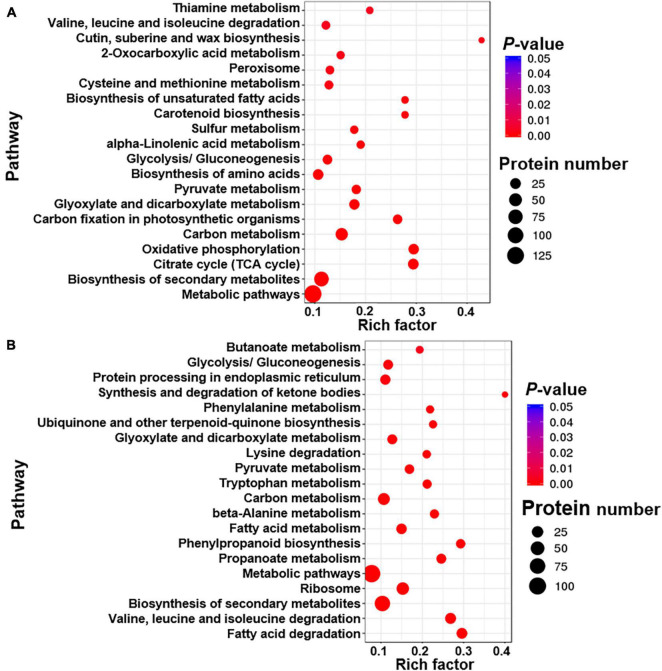
Top 20 enriched pathway terms of differentially expressed proteins in FS280 vs. WT. **(A)** Enriched pathways based on the upregulated proteins. **(B)** Enriched pathways based on the downregulated proteins. The rich factor is defined as the ratio of differentially expressed proteins to all proteins that were annotated in this pathway term. The dot size represents the number of differentially expressed proteins that were annotated to the pathway.

Differentially expressed proteins were defined as at least one replicate that was changed by more than 1.2-fold and had a *Q*-value less than 0.05 (Zhu et al., 2009; Zhang et al., 2015). Few DEPs were found between the two FS280 technical replicates (16 upregulated and 18 downregulated) and between the two WT technical replicates (3 upregulated and 2 downregulated) (Figure 4A), demonstrating the good reproducibility of the proteomic analysis. Good reproducibility was also indicated by the CV distribution (Figure 4B), showing that 88.20 and 98.00% of the identified proteins possessed a CV less than 10.00 and 20.00%, respectively. A total of 442 proteins were upregulated and 468 proteins were downregulated in FS280 compared with WT (Figure 4A and Supplementary Tables 1B,C). Against the KEGG database, all the DEPs were classified into 19 functional classifications in 5 branches of metabolic pathways (Supplementary Figure 3). Most of the DEPs (670, 73.63%) were classified into metabolic pathways, in which 105 (11.54%) DEPs, 88 (9.67%) DEPs, 58 (6.37%) DEPs, and 58 (6.37%) DEPs were classified into carbohydrate metabolism, amino acid metabolism, energy metabolism and lipid metabolism, respectively. The identified DEPs were further subjected to pathway functional enrichment analyses (Figure 5). The top 20 pathways with significant enrichment are shown in Figure 5, with the most enriched pathways of the upregulated DEPs in FS280 compared with WT, including “cutin, suberine and wax biosynthesis,” “oxidative phosphorylation,” “citrate cycle (TCA cycle),” “biosynthesis of unsaturated fatty acids,” “carotenoid biosynthesis,” “carbon fixation in photosynthetic organisms,” and “thiamine metabolism” (Figure 5A). Moreover, the most enriched pathways of the downregulated DEPs in FS280 compared with WT included “synthesis and degradation of ketone bodies,” “fatty acid degradation,” “phenylpropanoid biosynthesis,” “valine, leucine, and isoleucine degradation,” “propanoate metabolism,” “ubiquinone and other terpenoid-quinone biosynthesis,” and “phenylalanine metabolism” (Figure 5B).

##### Differentially Expressed Proteins Related to the Environmental Adaptation of Organismal Systems

As shown in Supplementary Figure 3, six (0.66%) DEPs induced by fermentation supernatant stress were identified as environmental adaptations of organismal systems, of which 3 proteins, calmodulin, calcium-dependent protein kinase, and calcium-binding protein CML (Gene. 133049:Unigene42234_All:g.133049:m.133049, Gene.120823:

Unigene35927_All:g.120823:m.120823, Gene.122831:Unigene 36887_All:g. 122831:m.122831), were upregulated by 1.337- to 1.594-fold and 3 other proteins, calcium-dependent protein kinase and heat shock protein 90 kDa beta (Gene.14362:CL1384.Contig1_All:g.14362:m.14362, Gene.3511: CL288. Contig4_All:g.3511:m.3511, Gene.70544:Unigene 9321_All:g.70544:m.70544), were downregulated by 0.648- to 0.845-fold (Supplementary Tables 1B,C). These 6 proteins (calmodulin, calcium-dependent protein kinase, calcium-binding protein CML, and heat shock protein 90 kDa beta) may be involved in the tolerance of the fermentation supernatant and were also found to be involved in stress tolerance in other studies (Chang et al., 2006; Campo et al., 2014; Jing et al., 2016; Wang et al., 2017). For example, high calmodulin concentrations were maintained in *Populus euphratica* under high salinity stress (Chang et al., 2006), and heat shock proteins were found to play important roles in both cold and heat tolerance of *Pichia pastoris* (Wang et al., 2017).

##### Differentially Expressed Proteins Related to the Citrate Cycle and Oxidative Phosphorylation

Dihydrolipoamide dehydrogenase, a component of the pyruvate dehydrogenase complex, was upregulated by 1.29-fold in FS280 (Table 1), which might provide more acetyl-CoA for the TCA cycle and lipid biosynthesis in FS280. The upregulated TCA cycle and oxidative phosphorylation pathways in FS280 would provide more substrates and energy for cell growth, consistent with the improved growth of FS280 by 161.87% compared with WT (Figures 1D, 5A). Comparative proteomics showed that 25 upregulated proteins in FS280 were involved in the TCA cycle (Supplementary Table 1B). For example, two proteins of citrate synthase in FS280 were significantly upregulated by 1.151- to 1.405-fold (Table 1). Five proteins of aconitate hydratase in FS280 were upregulated by 1.008- to 1.433-fold, and four proteins of malate dehydrogenase in FS280 were upregulated by 1.129- to 1.546-fold (Table 1). In addition, two subunits of succinyl-CoA synthetase and five subunits of succinate dehydrogenase in FS280 were upregulated by 1.150- to 1.323-fold and 1.007- to 1.308-fold, respectively (Table 1). At the same time, 23 proteins involved in oxidative phosphorylation were upregulated in FS280 (Supplementary Table 1B), including three subunits of ubiquinol-cytochrome c reductase, which were upregulated by 1.199- to 1.456-fold; eight subunits of V-type H+-transporting ATPase in FS280, which were upregulated by 1.114- to 1.559-fold; and seven subunits of F-type H+-transporting ATPase in FS280,which were upregulated by 1.050- to 1.498-fold (Supplementary Table 1B), suggesting the possible strengthened energy level in FS280.

**TABLE 1:** Differentially expressed proteins related to biosynthesis pathways in FS280 vs. WT.

Pathway	Function description (KEGG)	Protein ID	FS280-1/WT-1		FS280-2/WT-2		Up/down
			Ratio	*Q*-value	Ratio	*Q*-value	
Glycolysis	Glucose-6-phosphate isomerase	Gene.151366:Unigene51997_All:g.151366:m.151366	1.366	0.002	1.419	0.002	Up
	Fructose-bisphosphate aldolase	Gene.27708:CL3015.Contig1_All:g.27708:m.27708	1.089	0.230	1.250	0.002	Up
	Phosphoglycerate kinase	Gene.20997:CL2155.Contig7_All:g.20997:m.20997	1.277	0.133	1.409	0.002	Up
	Phosphoglycerate kinase	Gene.21010:CL2155.Contig13_All:g.21010:m.21010	1.217	0.002	1.408	0.002	Up
	Enolase	Gene.160751:Unigene64062_All:g.160751:m.160751	1.111	0.072	1.307	0.018	Up
	Pyruvate kinase	Gene.129809:Unigene41001_All:g.129809:m.129809	1.154	0.002	1.220	0.002	Up
Pyruvate decarboxylation	Dihydrolipoamide dehydrogenase	Gene.32846:CL3701.Contig3_All:g.32846:m.32846	1.308	0.002	1.265	0.002	Up
Citrate cycle	Citrate synthase	Gene.160348:Unigene63803_All:g.160348:m.160348	1.405	0.002	1.349	0.002	Up
	Citrate synthase	Gene.3504:CL286.Contig13_All:g.3504:m.3504	1.211	0.002	1.151	0.002	Up
	Aconitate hydratase	Gene.233:CL9.Contig4_All:g.233:m.233	1.125	0.219	1.251	0.008	Up
	Aconitate hydratase	Gene.137592:Unigene43924_All:g.137592:m.137592	1.008	0.936	1.201	0.021	Up
	Aconitate hydratase	Gene.154472:Unigene53462_All:g.154472:m.154472	1.122	0.081	1.433	0.002	Up
	Aconitate hydratase	Gene.240:CL9.Contig6_All:g.240:m.240	1.040	0.691	1.246	0.002	Up
	Aconitate hydratase	Gene.160292:Unigene63774_All:g.160292:m.160292	1.084	0.180	1.276	0.008	Up
	Succinyl-CoA synthetase alpha subunit	Gene.3372:CL283.Contig1_All:g.3372:m.3372	1.150	0.213	1.259	0.005	Up
	Succinyl-CoA synthetase alpha subunit	Gene.3386:CL283.Contig4_All:g.3386:m.3386	1.255	0.002	1.323	0.002	Up
	Succinate dehydrogenase (ubiquinone) flavoprotein subunit	Gene.4358:CL354.Contig14_All:g.4358:m.4358	0.883	0.148	1.235	0.005	Up
	Succinate dehydrogenase (ubiquinone) flavoprotein subunit	Gene.4323:CL354.Contig6_All:g.4323:m.4323	1.157	0.049	1.290	0.002	Up
	Succinate dehydrogenase (ubiquinone) iron-sulfur subunit	Gene.48371:CL6104.Contig4_All:g.48371:m.48371	1.308	0.320	1.205	0.041	Up
	Succinate dehydrogenase (ubiquinone) iron-sulfur subunit	Gene.48369:CL6104.Contig3_All:g.48369:m.48369	1.007	0.952	1.247	0.002	Up
	Succinate dehydrogenase (ubiquinone) iron-sulfur subunit	Gene.48368:CL6104.Contig2_All:g.48368:m.48368	1.127	0.099	1.278	0.002	Up
	Malate dehydrogenase	Gene.46910:CL5873.Contig2_All:g.46910:m.46910	1.129	0.002	1.292	0.002	Up
	Malate dehydrogenase	Gene.7390:CL663.Contig4_All:g.7390:m.7390	1.546	0.003	1.428	0.003	Up
	Malate dehydrogenase	Gene.46914:CL5873.Contig3_All:g.46914:m.46914	1.170	0.002	1.266	0.002	Up
	Malate dehydrogenase	Gene.72616:Unigene10324_All:g.72616:m.72616	1.249	0.003	1.163	0.043	Up
Biosynthesis of unsaturated fatty acids	Acetyl-CoA acyltransferase 1	Gene.160568:Unigene63932_All:g.160568:m.160568	1.292	0.002	1.274	0.002	Up
	Acetyl-CoA acyltransferase 1	Gene.9109:CL841.Contig10_All:g.9109:m.9109	1.391	0.002	1.257	0.092	Up
	Acetyl-CoA acyltransferase 1	Gene.9079:CL841.Contig3_All:g.9079:m.9079	1.168	0.002	1.273	0.002	Up
	Acetyl-CoA acyltransferase 1	Gene.9102:CL841.Contig9_All:g.9102:m.9102	1.296	0.002	1.389	0.002	Up
	Acyl-coenzyme A thioesterase 1/2/4	Gene.129372:Unigene40838_All:g.129372:m.129372	1.320	0.002	1.101	0.276	Up
Fatty acid biosynthesis	Medium-chain acyl-[acyl-carrier-protein] hydrolase	Gene.7220:CL645.Contig1_All:g.7220:m.7220	1.226	0.002	1.203	0.002	Up
Fatty acid degradation	Enoyl-CoA hydratase	Gene.6829:CL605.Contig2_All:g.6829:m.6829	0.919	0.522	0.772	0.002	Down
	Enoyl-CoA hydratase	Gene.72545:Unigene10297_All:g.72545:m.72545	0.828	0.002	0.841	0.006	Down
	Enoyl-CoA hydratase	Gene.6832:CL605.Contig3_All:g.6832:m.6832	0.894	0.005	0.826	0.002	Down
	Acyl-CoA dehydrogenase	Gene.10853:CL1004.Contig1_All:g.10853:m.10853	0.936	0.877	0.748	0.005	Down
	Acyl-CoA dehydrogenase	Gene.10873:CL1004.Contig6_All:g.10873:m.10873	0.728	0.240	0.696	0.046	Down
	Acyl-CoA dehydrogenase	Gene.88805:Unigene19403_All:g.88805:m.88805	0.727	0.002	0.733	0.003	Down
	Acyl-CoA dehydrogenase	Gene.58517:Unigene3441_All:g.58517:m.58517	0.726	0.143	0.663	0.018	Down
	Acyl-CoA dehydrogenase	Gene.80910:Unigene14679_All:g.80910:m.80910	0.831	0.030	0.809	0.002	Down
	Acyl-CoA dehydrogenase	Gene.110939:Unigene30664_All:g.110939:m.110939	0.759	0.002	0.685	0.002	Down
	Long-chain acyl-CoA synthetase	Gene.80103:Unigene14507_All:g.80103:m.80103	0.832	0.002	0.873	0.002	Down
	Acetyl-CoA C-acetyltransferase	Gene.8959:CL826.Contig5_All:g.8959:m.8959	0.770	0.303	0.663	0.042	Down
	Acetyl-CoA C-acetyltransferase	Gene.69434:Unigene9069_All:g.69434:m.69434	0.792	0.002	0.836	0.005	Down
	Acetyl-CoA C-acetyltransferase	Gene.13769:CL1315.Contig2_All:g.13769:m.13769	0.733	0.031	0.743	0.010	Down
	Acetyl-CoA C-acetyltransferase	Gene.13764:CL1315.Contig1_All:g.13764:m.13764	0.710	0.030	0.659	0.005	Down
	Long-chain-fatty-acid–CoA ligase ACSBG	Gene.110188:Unigene30515_All:g.110188:m.110188	0.867	0.007	0.817	0.002	Down
Starch biosynthesis	Starch synthase	Gene.120642:Unigene35897_All:g.120642:m.120642	0.653	0.002	0.669	0.002	Down
	1,4-alpha-glucan branching enzyme	Gene.2141:CL165.Contig3_All:g.2141:m.2141	0.833	0.005	0.815	0.009	Down
Starch degradation	Trehalose 6-phosphate synthase/phosphatase	Gene.25225:CL2690.Contig3_All:g.25225:m.25225	1.484	0.006	1.900	0.002	Up
β-Carotene degradation	Beta-Carotene isomerase	Gene.134342:Unigene42788_All:g.134342:m.134342	1.593	0.059	1.973	0.042	Up
	Beta-Ring hydroxylase	Gene.90965:Unigene20025_All:g.90965:m.90965	1.296	0.002	1.260	0.010	Up
	Abscisic-aldehyde oxidase	Gene.13944:CL1327.Contig2_All:g.13944:m.13944	1.284	0.002	1.378	0.002	Up
	Abscisic-aldehyde oxidase	Gene.13948:CL1327.Contig3_All:g.13948:m.13948	1.160	0.182	1.371	0.003	Up
	(+)-Abscisic acid 8’-hydroxylase	Gene.58344:Unigene3389_All:g.58344:m.58344	1.601	0.002	1.560	0.002	Up

##### Differentially Expressed Proteins Related to Glycolysis/Gluconeogenesis

The glycolysis/gluconeogenesis pathway was among the upregulated enriched pathways with a rich factor > 0.1 (Figure 5A), and a total of 16 proteins related to glycolysis/gluconeogenesis were identified as upregulated (Supplementary Table 1B). For example, pyruvate kinase, which is involved in one of the rate-limiting steps of glycolysis metabolism, was upregulated by 1.190-fold in FS280 compared to WT (Table 1). Glucose-6-phosphate isomerase and fructose-bisphosphate aldolase were upregulated by 1.390- and 1.170-fold, respectively (Table 1). Two proteins of phosphoglycerate kinase were upregulated by 1.310- and 1.340 -fold, and the enolase was upregulated by 1.210-fold (Table 1). The strengthened glycolysis pathway could provide energy support for the growth of FS280, which contributed to the growth of FS280 that was increased by 161.87% at 72 h in the presence of 90% (*v/v*) fermentation supernatant compared to WT and provided more substrates for the lipid biosynthesis in FS280.

##### Differentially Expressed Proteins Related to the Biosynthesis of Lipids

Comparative proteomics analysis revealed that four proteins of acetyl-CoA acyltransferase 1 (1.168- to 1.391-fold) and one acyl-coenzyme A thioesterase 1/2/4 (1.210-fold) involved in the biosynthesis of unsaturated fatty acids were upregulated in FS280 compared to WT (Table 1 and Figure 5A). In addition, medium-chain acyl-[acyl-carrier-protein] hydrolase, which is involved in fatty acid biosynthesis, was upregulated by 1.210-fold in FS280 compared to WT (Table 1). These results might partially explain why the DHA in DCW was significantly increased in FS280 compared with WT (Figure 2B). Moreover, the pathways of cutin, suberine and wax biosynthesis were also upregulated in FS280 compared with WT (Figure 5A), consistent with the significant increase in lipids in DCW in FS280 compared with WT (Figure 2B). Previous studies showed that cutin, suberine and wax could protect cells against stress and pathogen attack (Panikashvili et al., 2010; Lee and Suh, 2013; Gou et al., 2017). Upregulated cutin, suberine and wax biosynthesis might increase the lipid content of FS280 compared with that of WT as well as increase the stress tolerance to fermentation supernatant in FS280.

##### Differentially Expressed Proteins Related to Fatty Acid Degradation

Fatty acid degradation was identified as one of the most enriched pathways in FS280 for the downregulated proteins (Figure 5B). A total of 21 proteins involved in fatty acid degradation were identified to be downregulated in FS280 compared with WT in the proteomic analysis (Supplementary Table 1C), including long-chain acyl-CoA synthetase and long-chain-fatty-acid–CoA ligase ACSBG, which were downregulated by 0.850- and 0.840-fold, respectively, and acyl-CoA dehydrogenase, which was downregulated by 0.663- to 0.936-fold (Table 1). Furthermore, enoyl-CoA hydratase was downregulated by 0.772- to 0.919-fold, and acetyl-CoA C-acetyltransferase was downregulated by 0.659- to 0.836-fold (Table 1). To improve the lipid content of cells, most studies have focused on fatty acid biosynthesis, whereas few studies have focused on fatty acid degradation (Pei et al., 2017; Bi et al., 2018). This study demonstrated that the attenuation of fatty acid degradation might be important for increasing lipid content in *C. cohnii*.

##### Differentially Expressed Proteins Related to Starch Biosynthesis and Degradation

Notably, starch synthase and 1,4-alpha-glucan branching enzymes involved in starch biosynthesis were downregulated in FS280 compared with WT by 0.660- and 0.820-fold, respectively, while trehalose 6-phosphate synthase/phosphatase involved in starch degradation was upregulated by 1.690-fold (Table 1), which was consistent with the reduced content of total starches in FS280 and was only 38.23% of the starch content in WT (Figure 2B). These results indicated that the carbon sources in FS280 might be redirected from starch biosynthesis to lipid biosynthesis, both of which are the main storage compounds in *C. cohnii* competing for glucose utilization (Diao et al., 2019). It has been demonstrated that the lipid content could be increased significantly by reducing the starch content in many microalgae, including *C. cohnii* (Li et al., 2010; Diao et al., 2019). For example, triacylglycerol was increased by 10-fold in a *Chlamydomonas* starchless mutant that was defective in ADP-glucose pyrophosphorylase (Li et al., 2010).

##### Differentially Expressed Proteins Related to Carotene Degradation and Amino Acid Metabolism

γ-Carotene and β-carotene are the major carotenoids in *C. cohnii* (Withers and Tuttle, 1979; Sun et al., 2019). The results showed that several upregulated proteins were involved in γ-carotene and β-carotene degradation (Figure 5A and Table 1), which might be responsible for the decreased carotenoid contents in the evolved FS280 (Figure 2B). For example, β-carotene isomerase, β-ring hydroxylase and (+)-abscisic acid 8’-hydroxylase were upregulated in FS280 compared with WT by 1.780-, 1.280-, and 1.580-fold, respectively. In addition, abscisic-aldehyde oxidase was upregulated in FS280 compared with WT by 1.160- to 1.378-fold.

The biosynthesis of amino acids was upregulated in FS280 compared with WT (Figure 5A). A total of 24 upregulated proteins were found to be related to the biosynthesis of amino acids (Supplementary Table 1B), which was consistent with the fast consumption of ammonium nitrogen in FS280 (Figure 2A). The pathways of valine, leucine, isoleucine and lysine degradation were downregulated in FS280 compared with WT (Figure 5B). The upregulated amino acid and fatty acid degradation in WT suggested that *C. cohnii* might provide more energy for growth by degrading fatty acids and amino acids under fermentation supernatant stress conditions.

**FIGURE 6:**
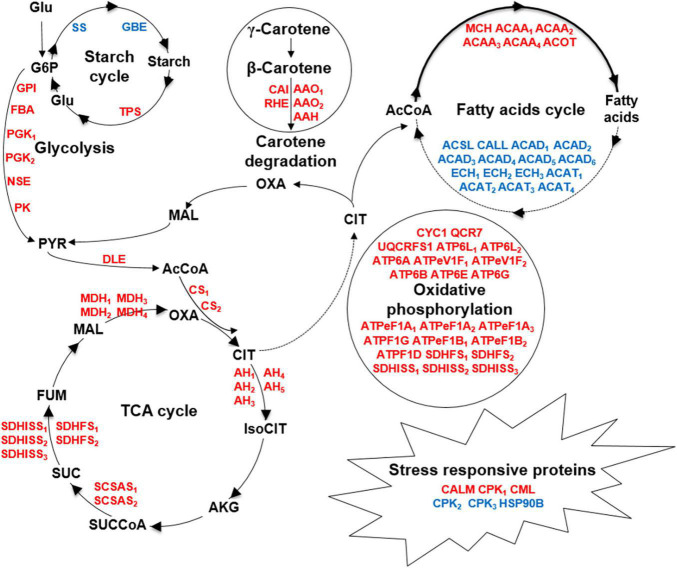
Comparative metabolic responses to fermentation supernatant stress between FS280 and WT. Upregulated and downregulated proteins are indicated by red and blue, respectively. Glu, glucose; G6P, glucose-6-phosphate; PYR, pyruvate; AcCoA, acetyl-CoA; CIT, citrate; IsoCIT, isocitrate; AKG, α-ketoglutarate; SUCCoA, succinyl-CoA; SUC, succinate; FUM, fumarate; MAL, malate; OXA, oxaloacetate; GPI, glucose-6-phosphate isomerase; FBA, fructose-bisphosphate aldolase; PGK, phosphoglycerate kinase; NSE, enolase; PK, pyruvate kinase; DLE, dihydrolipoamide dehydrogenase; CS, citrate synthase; AH, aconitate hydratase; SCSAS, succinyl-CoA synthetase alpha subunit; SDHFS, succinate dehydrogenase (ubiquinone) flavoprotein subunit; SDHISS, succinate dehydrogenase (ubiquinone) iron-sulfur subunit; MDH, malate dehydrogenase; ACAA, acetyl-CoA acyltransferase 1; ACOT, acyl-coenzyme A thioesterase 1/2/4; MCH, medium-chain acyl-[acyl-carrier-protein] hydrolase; ECH, enoyl-CoA hydratase; ACAD, acyl-CoA dehydrogenase; ACSL, long-chain acyl-CoA synthetase; ACAT, acetyl-CoA C-acetyltransferase; CALL, long-chain-fatty-acid–CoA ligase ACSBG; SS, starch synthase; GBE, 1,4-alpha-glucan branching enzyme; TPS, trehalose 6-phosphate synthase/phosphatase; CAI, beta-carotene isomerase; RHE, beta-ring hydroxylase; AAO, abscisic-aldehyde oxidase; AAH, (+)-abscisic acid 8’-hydroxylase, CALM, calmodulin; CPK, calcium-dependent protein kinase; CML, calcium-binding protein CML; HSP90B, heat shock protein 90 kDa beta; CYC1, ubiquinol-cytochrome c reductase cytochrome c1 subunit; QCR7, ubiquinol-cytochrome c reductase subunit 7; ATP6L, V-type H+-transporting ATPase 16 kDa proteolipid subunit; ATP6A, V-type H+-transporting ATPase subunit A; ATPeV1F, V-type H+-transporting ATPase subunit F; ATP6B, V-type H+-transporting ATPase subunit B; ATP6E, V-type H+-transporting ATPase subunit E; ATP6G, V-type H+-transporting ATPase subunit G; ATPeF1A, F-type H+-transporting ATPase subunit alpha; ATPF1G, F-type H+-transporting ATPase subunit gamma; ATPeF1B, F-type H+-transporting ATPase subunit beta; ATPF1D, F-type H+-transporting ATPase subunit delta.

To our knowledge, this is the first proteomic approach applied to *C. cohnii*, which helped to identify the proteins that responded to fermentation supernatant stress and reveal the metabolic network change between the evolved strain FS280 and WT under fermentation supernatant stress (Figure 6). This study demonstrated that the upregulated pathways in FS280, including glycolysis, the TCA cycle, oxidative phosphorylation, and biosynthesis of amino acids, might contribute to its improved growth (Figures 5, 6) and that the upregulated biosynthesis of fatty acids and other lipids, starch and carotene degradation, and downregulated fatty acid degradation and starch biosynthesis might contribute to the increase lipid content of the evolved strain FS280 compared to WT (Figures 5, 6). In addition, as previous studies have usually focused on the synthesis of fatty acids, with little attention given to the degradation of fatty acids (Pei et al., 2017), and the relationship between fatty acid accumulation and starch or carotene (Diao et al., 2019; Sun et al., 2019), our study suggested that the degradation of fatty acids in *C. cohnii* might also be important for fatty acid accumulation and that the accumulation of starch and carotenoids might play a synergistic role in lipid accumulation in *C. cohnii*. This study provided an effective strategy to solve the bottleneck of the inhibitory fermentation supernatant and provided valuable potential targets for further improving growth and lipid accumulation via genetic engineering of *C. cohnii* and other oil-bearing microalgae.

## Data Availability Statement

The datasets presented in this study can be found in online repositories. The names of the repository/repositories and accession number(s) can be found in the article/Supplementary Material.

## Author Contributions

LC and WZ conceived and designed the study. LL and LZ performed the experiments. LL, JD, YB, LZ, FW, LC, and WZ analyzed the data and wrote the manuscript. All authors read and approved the manuscript.

## Conflict of Interest

The authors declare that the research was conducted in the absence of any commercial or financial relationships that could be construed as a potential conflict of interest.

## Publisher’s Note

All claims expressed in this article are solely those of the authors and do not necessarily represent those of their affiliated organizations, or those of the publisher, the editors and the reviewers. Any product that may be evaluated in this article, or claim that may be made by its manufacturer, is not guaranteed or endorsed by the publisher.

## Footnotes

1
http://proteomecentral.proteomexchange.org

## Supplementary Material

Supplementary Figure 1 Distribution of the identified proteins in *C. cohnii*. **(A)** Distribution of unique peptides. **(B)** Distribution of protein coverage. **(C)** Distribution of protein mass.

Supplementary Figure 2 Pathway annotation for all the identified proteins.

Supplementary Figure 3 Classification statistics of the pathways for differentially expressed proteins in FS280 vs. WT.
