# Supplementary material for: Rewiring the Metabolic Network to Increase Docosahexaenoic Acid Productivity in Crypthecodinium cohnii by Fermentation Supernatant-Based Adaptive Laboratory Evolution
Source: Front Microbiol. 2022 Mar 2;13:824189. doi: 10.3389/fmicb.2022.824189 (PMC8924677; doi:10.3389/fmicb.2022.824189)
Supplement: Supplementary Figure 1 — Distribution of the identified proteins in C. cohnii. (A) Distribution of unique peptides. (B) Distribution of protein coverage. (C) Distribution of protein mass. [file Presentation_1.PPTX]

## Slide 1
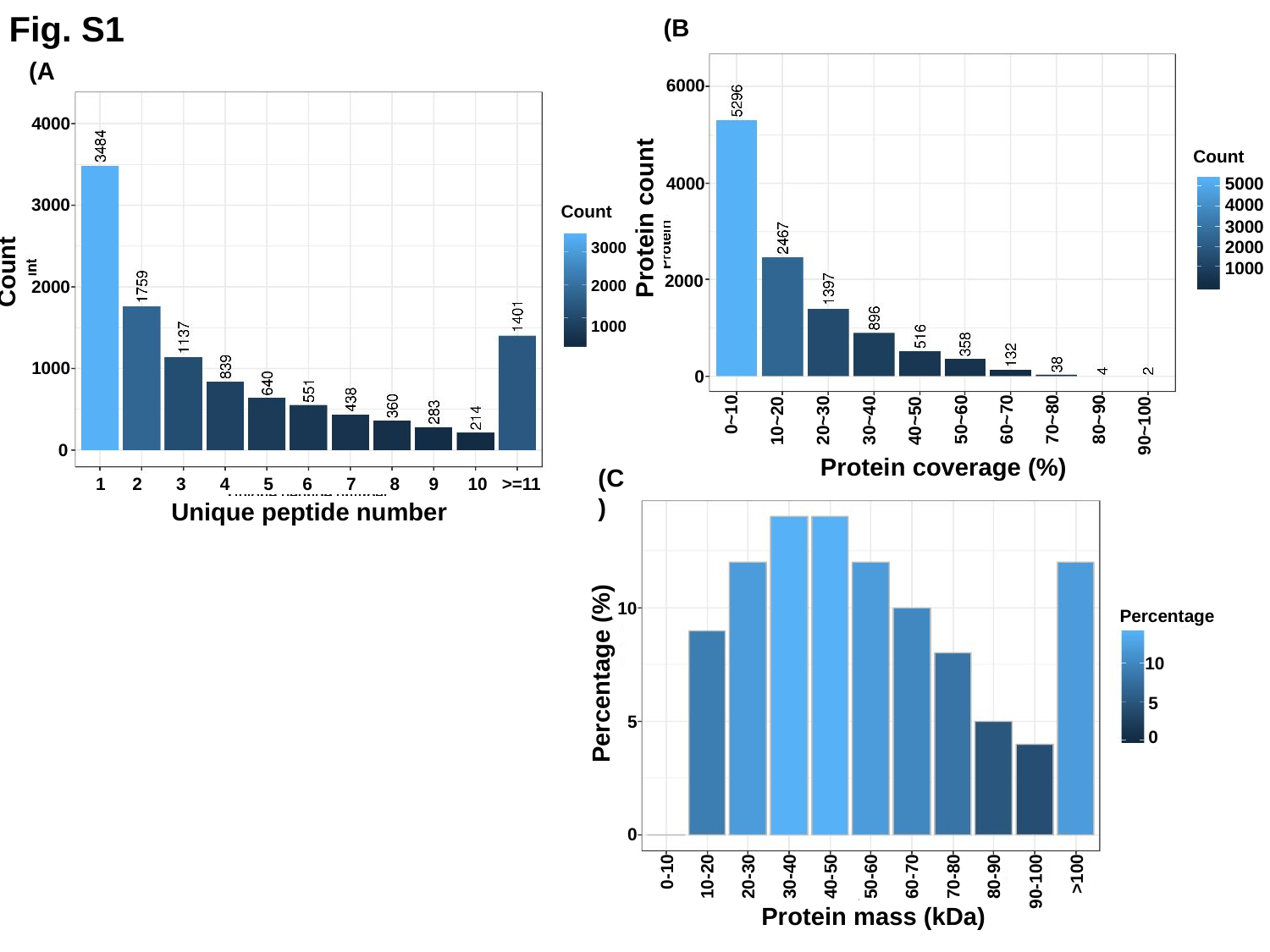

Fig. S1
(B)
Protein count
6000
Count
4000
5000
4000
3000
2000
1000
2000
0
60~70
70~80
80~90
0~10
50~60
20~30
30~40
10~20
40~50
90~100
Protein coverage (%)
(A)
4000
Count
3000
2000
1000
 2 3 4 5 6 7 8 9 10 >=11
Count
3000
2000
1000
0
Unique peptide number
(C)
Percentage (%)
10
Percentage
10
5
5
0
0
0-10
10-20
20-30
30-40
40-50
50-60
60-70
70-80
80-90
90-100
>100
Protein mass (kDa)

## Slide 2
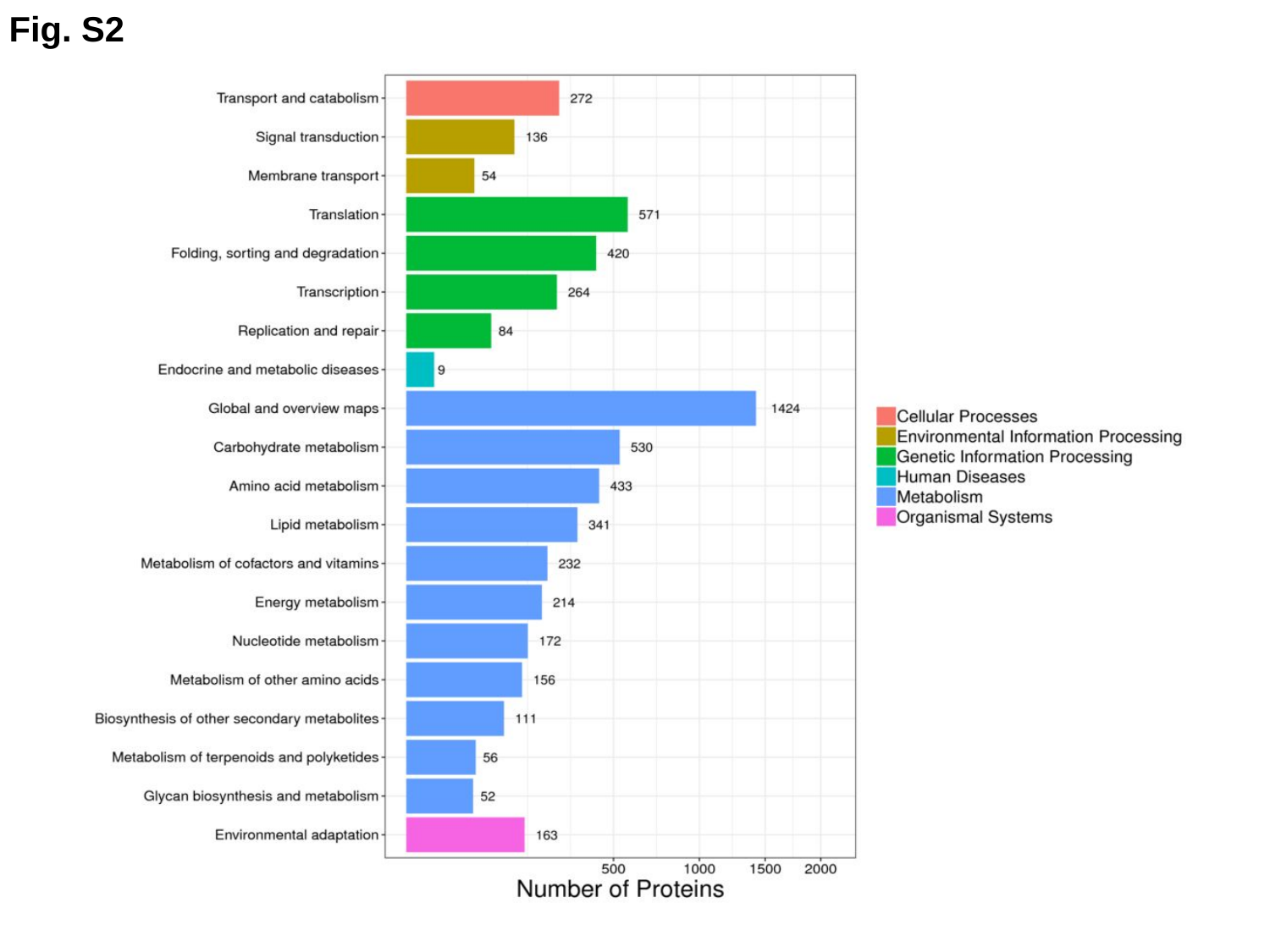

Fig. S2

## Slide 3
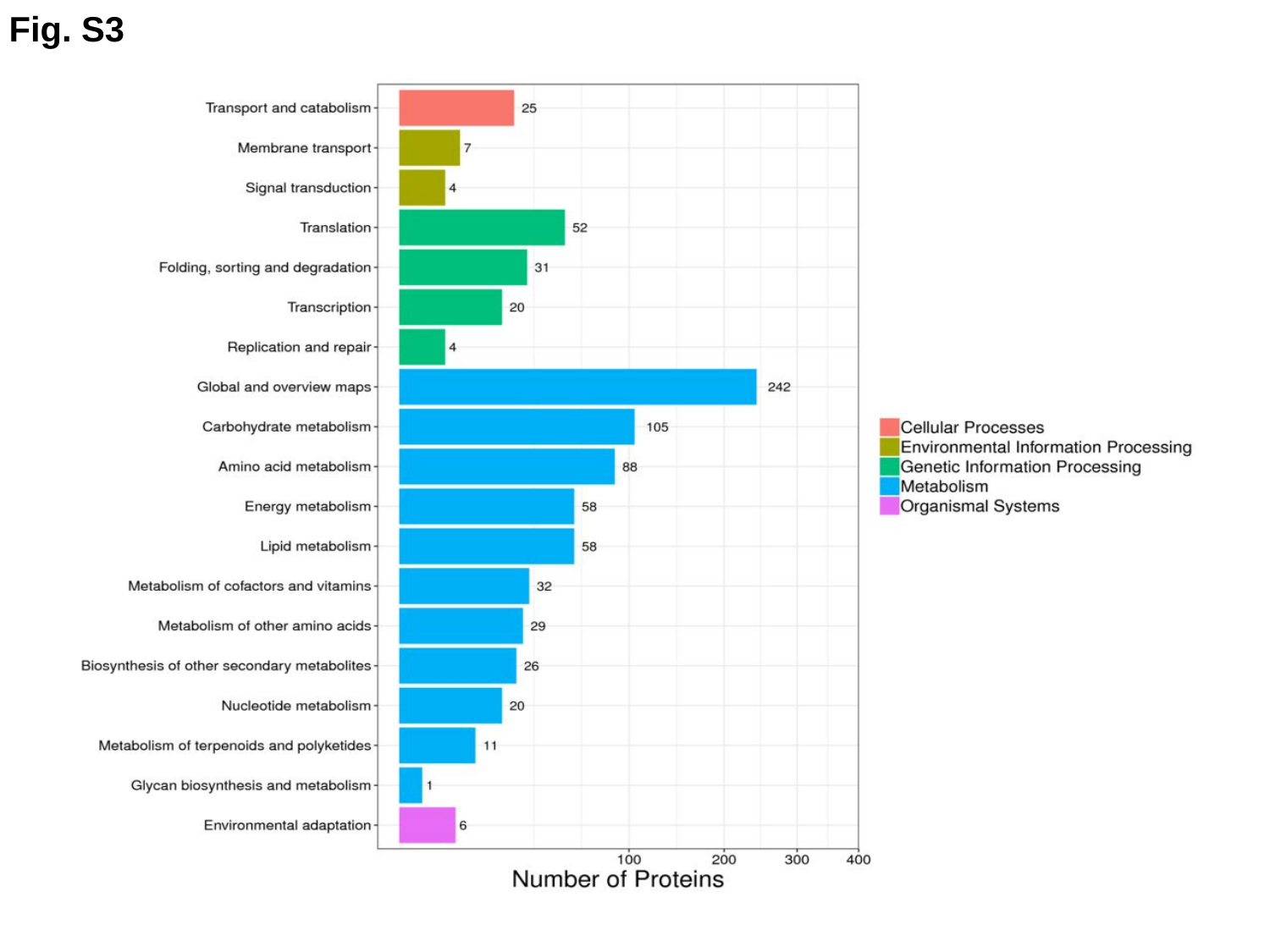

Fig. S3
